# HOW CAN WE ADVANCE THE FIELD OF GERONTOLOGY BY LEVERAGING OUR LIVED EXPERIENCES AS GERONTOLOGISTS AND GERIATRICIANS?

**DOI:** 10.1093/geroni/igad104.1103

**Published:** 2023-12-21

**Authors:** Laura Donorfio

**Affiliations:** University of Connecticut, Waterbury, Connecticut, United States

## Abstract

Reflecting on my development as a professor of gerontology has become a normative part of my mid-life career. Through this reflection, I have identified 4 stages of career progression, none of which were planned nor anticipated. I have labeled the stages: 1. Content and Logistics; 2. Confidence and Innovation; 3. Generativity and Meaning; and 4. Being the Other. This last stage, which I presently float in and out of, has made me very aware that my teaching about aging has intersected with my personal aging and unexpectedly, has shaped my personal pedagogy in profound ways--increased empathy, sensitivity, and social awareness. I am living the subject matter I thought I intimately knew. I am the other. Has my teaching been inauthentic? Hypocritical? Ageist? Aging and ageism are now personal. Others have written about this mental transition or awakening (Brody, 2010; Cohen et al., 2022; Greenberg, 2022; Pruchno, 2017; Scheidt, 2017), but very little scholarship has been directed towards this phenomenon. O’Neill (2016) also brings attention to this shares, “Although geriatric medicine is widely perceived as an advocate for older people, it is striking how little scholarship has been directed towards the attitudes and personal beliefs of geriatricians toward ageing and, in particular, towards their own future narratives.” This presentation will draw from the existing literature to frame this emergent 4th stage of career progression and begin the discussion about how this insight can be leveraged to advance the fields of gerontology/geriatrics and teach the next generation of scholars.

